# Behavioral evidence suggests the presence of a female-emitted sex pheromone in the water mite species, *Arrenurus globator* (O. F. Muller, 1776) (Acari: Hydrachnida; Arrenuridae)

**DOI:** 10.1007/s10493-025-01009-7

**Published:** 2025-02-26

**Authors:** Mariusz Więcek, Michal Knapp, Grażyna Greczka

**Affiliations:** 1 Department of Ecology, Faculty of Environmental Sciences, Czech University of Life Sciences Prague, Kamýcká 129, Prague, Suchdol 165 00 Czech Republic; 2 Department of Computer Science and Statistics, Poznan University of Medical Sciences, Rokietnicka 7, Poznań, 60-806 Poland; 3 Department of Otolaryngology and Head and Neck Surgery, Poznan University of Medical Sciences, Przybyszewskiego 49, Poznań, 60-355 Poland

**Keywords:** Courtship, Chemoreception, Chemical communication, Mating behavior, Semiochemicals

## Abstract

Species from various animal taxa have been found to use pheromonal communication underwater. Although the use of pheromones in water mites has been previously suggested, experimental evidence for it remains sparse. We examined the behavioral responses of male and female *Arrenurus globator* to water in which conspecifics of the same and the opposite sex had been kept, in order to test the hypothesis that chemical communication occurs between sexes. Results suggest a putative female-produced sex pheromone that stimulates at least the initial steps of mating behavior in males. Males exhibited arrestant behavior, leg fanning and readiness posture more in female-conditioned water than in male-conditioned or control water. In contrast, females showed no response to either male-conditioned water or female-conditioned water.

## Introduction

Pheromonal communication has been documented in various aquatic taxa, including Crustacea, Gastropoda, Cephalopoda, Polychaeta, and Echinodermata (Breithaupt and Hardege 2012; Derby 2020). Similarly, the use of different types of sex pheromones has been frequently suggested across various phylogenetic lineages of mites (Sonenshine 1985; Krantz and Walter 2009). Water mites use both mechano- and chemoreception for prey selection and mating behavior (Baker 1996). However, most evidence for sex pheromones in water mites relies on observations that have not been experimentally verified. For example, male *Neumania papillator* Marshall, 1922, have been observed fanning their legs, presumably sending a pheromone-laden current of water from the spermatophores to nearby females (Proctor 1991). It is likely that spermatophore-associated pheromones are, at the very least, widespread in various water mite groups (Proctor 1991, 1992).

The genus *Arrenurus* Dugès, 1834, with approximately 1,000 described species, is the most diverse genus of water mites (Smit 2020). *Arrenurus globator* (O. F. Müller, 1776) is a common species that occurs in both standing and flowing freshwater habitats throughout the Palearctic (Böttger 1962; Gerecke et al. 2016). Adults and deutonymphs of *A. globator* are predators that primarily feed on Ostracoda, while the larvae parasitize a wide range of aquatic insects, including Diptera and Coleoptera (e.g., Böttger 1962; Böttger and Martin 2003; Gerecke et al. 2016). Importantly, the species status of *A. globator* was confirmed in recent years through the use of morphology and molecular markers (e.g., Więcek et al. 2023).

**Fig. 1:**
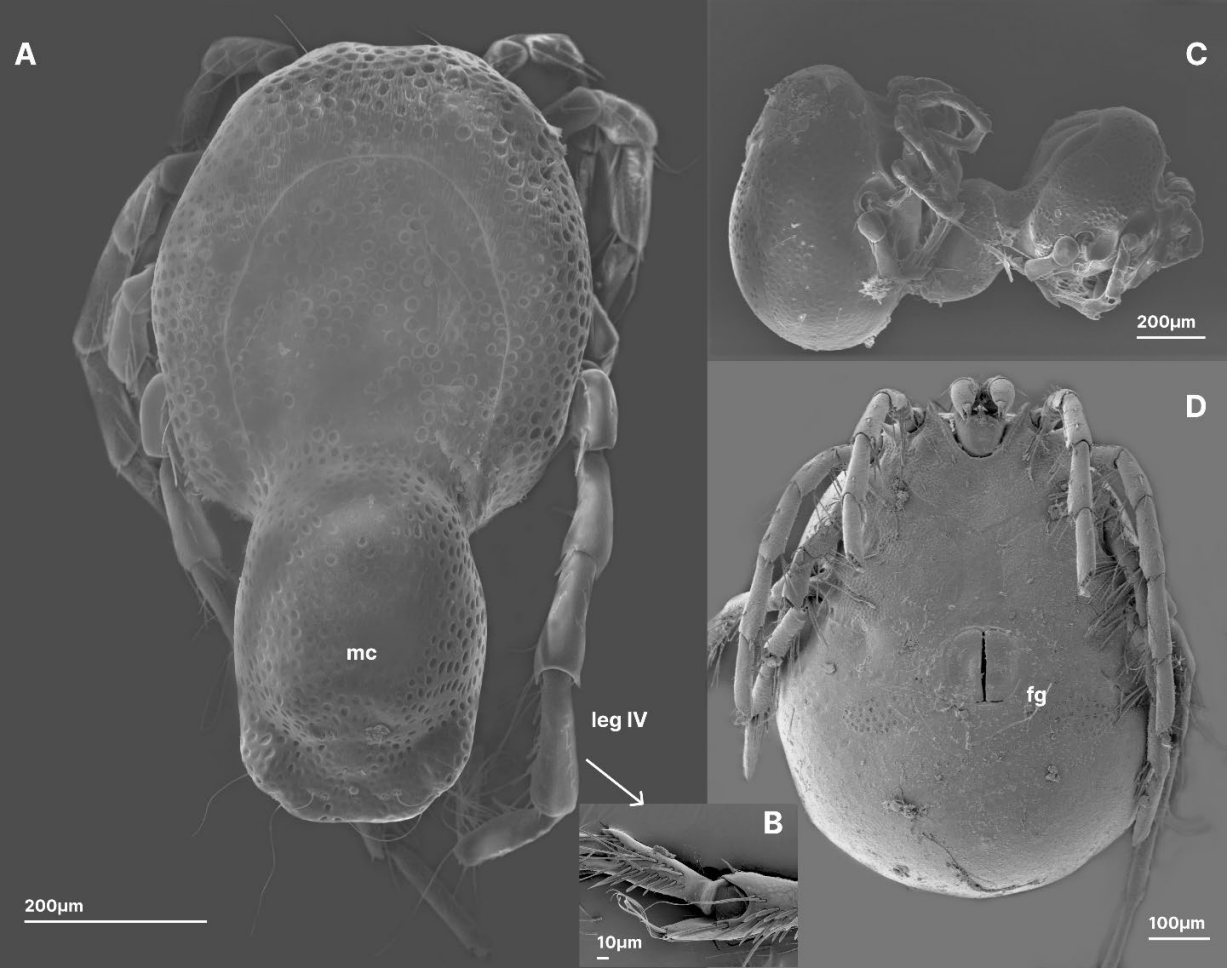
Scanning electron microscope images of *Arrenurus globator*: **A**– male, dorsal view (note the elongated and modified cauda), **B**– male, fourth and fifth segments of leg IV involved in the leg fanning behaviour; **C** - male and female in copula (lateral view); female glued to male’s cauda; **D**– female, ventral view; Abbreviations: mc = male’s cauda, fg = female’s genital opening; (after Więcek 2016; modified)

The mating behavior of *A. globator*, as well as many other representatives of the genus, is complex. During the initial mating phase, the male stops swimming, fans his hind legs, and, when the female is nearby, places his fourth legs flat over his hind body (cauda). In the later stages of mating, a male with a female glued to his cauda exhibits more elaborate behaviors, such as rocking and vigorous jerking of the cauda (Böttger 1962; Więcek 2016) (see Fig. 1 for SEM images of morphological structures involved in courtship behavior). In the pre-pairing stage of mating, females typically walk and move their fourth legs in a rotary motion. In close proximity to a male, they may stop walking and climb onto the male’s cauda. Once glued to the male’s cauda, the male maneuvers the female until sperm transfer is completed (Więcek 2016). Although it has been postulated that *A. globator* males and females can sense each other at a distance, there is no conclusive evidence of chemical communication between potential mates (Böttger 1962).

As far as the authors are aware, only three studies have used controlled experiments to investigate communication through sex pheromones in *Arrenurus* mites. Baker (1996) reported that female *Arrenurus acutus* (Marshall, 1908) were attracted to males, with the attraction decreasing with distance. Smith and Hagman (2002) identified a non-polar compound produced by female *Arrenurus manubriator* (Marshall, 1903) that elicited a behavioral response in males. Additionally, cross-attractancy between species has been demonstrated in *Arrenurus*, indicating the potential for interspecific communication in this genus (Smith and Florentino 2004).

In our study, we used behavioral observations of male and female responses to water in which conspecifics had been kept, with the aims of: (a) determining whether there is chemical communication between the sexes in *A. globator* and (b) identifying behaviors displayed by males and females that are likely related to the reception or dispersal of potential sex pheromones. We compared our results with the existing knowledge of communication mediated by sex pheromones in other water mite species.

## Materials and methods

### Mite collection and maintenance

The mites used in this study were collected from a shallow pond overgrown with dense submerged vegetation (Milíčovský rybník; Prague, Czech Republic; 50°01′33.3″N 14°32′22.5″E) in late July and early August 2023. The samples were collected using a hand net with a mesh size of 250 μm. Field-collected adults (a mixture of ages) were sorted by sex into two separate 80 ml glass containers, each holding 130 individuals. The mites were kept in tap water (aged for at least 24 h) at room temperature (~ 24 °C) under the natural day/night rhythm for 74 h. They were not fed during this period to exclude the presence of kairomones produced by food organisms. The female- and male-conditioned water obtained in this way was then used in the experiments. The tap water used as a control was kept in a glass container under the same conditions and for the same duration as the water in which the mites were stored.

### Experimental methods

We applied modified experimental designs of the experiments on pheromonal communication among *Arrenurus* species described by Smith and Hagman (2002) and Smith and Florentino (2004). In all experiments, the test arena was a glass Petri dish (5 cm in diameter, 1.5 cm in height) filled with 2.5 ml of water and divided into two equal parts by a line painted on the bottom. Two 2 ml plastic syringes were attached to a stand on opposite sides of the arena, with their needle tips positioned below the water surface and 0.5 cm from the dish edge. One syringe contained female- or male-conditioned water, while the other contained control water.

Before testing mite responses, we assessed whether conditioned water and control water introduced on opposite sides of the test arena would mix during the experiment. For this, we used the same setup later applied to test behavioral responses. Water colored with potassium permanganate (0.1 g per 1000 cm³) and clear tap water were simultaneously introduced via gravitational flow on opposite sides of the dish. In twenty repetitions, the colored water did not penetrate beyond the central line at the bottom of the Petri dish during approximately 1 min of syringe emptying.

Behavioral observations began immediately after a single mite was introduced into the center of the arena. The plungers of the syringes were pulled out simultaneously, releasing 1 ml of control water and 1 ml of treated water on opposite sides of the dish. The mites’ behavior was observed with a magnifying glass with 10x magnification during the water’s gravitational flow (~ 1 min). Experiments were conducted at room temperature (~ 24 °C) in daylight, with additional illumination from a LED lamp (approx. 6500 K). Each specimen was observed in a new dish. Some dishes were reused after washing with tap water and drying at ~ 50 °C. Notably, residual compound(s) oxidise when dried unless desiccated under nitrogen, and tested mites show no differences in behavioral activity when exposed to dry-treated Petri dishes (Bruce Smith; pers. comm.).

For males, we scored three behaviors: (a) arrestant behavior = male stops swimming and remains motionless, (b) readiness posture (leg-crook behavior) = male crooks his hind legs at the fourth segment and places them over his cauda and (c) leg fanning = male moves his hind legs in a rotary motion. We considered the occurrence of one or more of the above behaviors within the half of the dish where conditioned water was introduced as a positive reaction, while the lack of behavioral response (or a response on the control side) was considered a negative reaction. For females, we used the same approach, scoring the same behaviors except for the readiness posture, which is displayed only by males (Böttger 1962; Więcek 2016). Each individual was tested separately and used once.

**Fig. 2:**
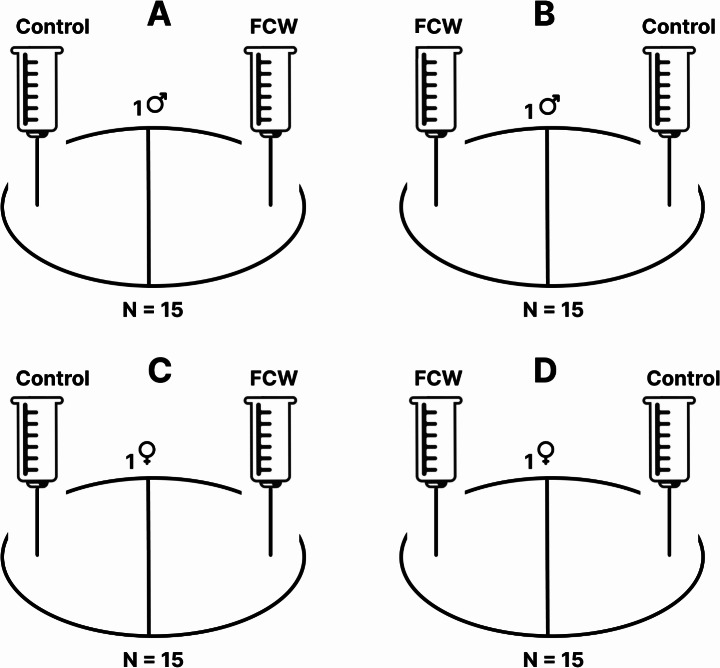
Schematic representation of the experimental setups; **A**, **B** - experiment no. 1, reactions of males exposed to female-conditioned water and control water, **C**, **D** - experiment no. 2, reactions of females exposed to female-conditioned water and control water. Female-conditioned water was added to the right side of the test arena while control water was introduced to the left side (*N* = 15). In the second setup (*N* = 15), the arrangement was reversed. Males and females were tested individually. FCW– female-conditioned water

**Fig. 3:**
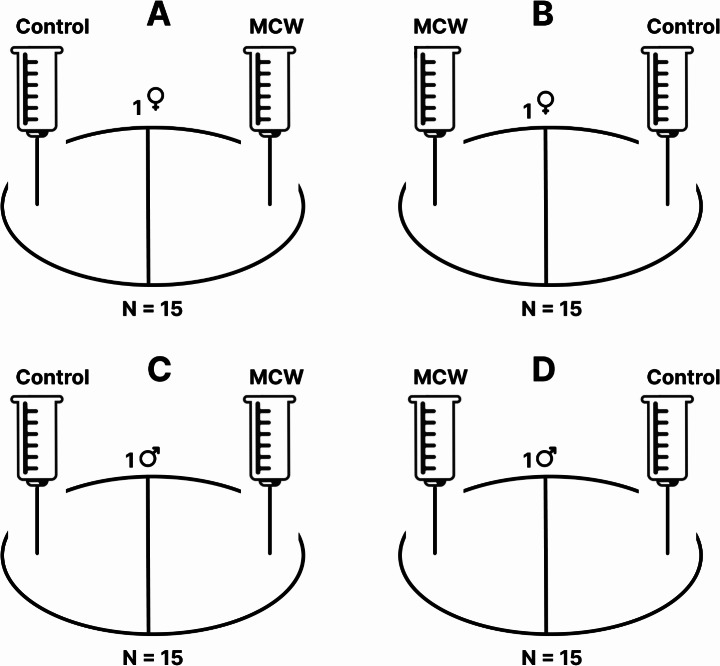
Schematic representation of the experimental setups; **A**, **B** - experiment no. 3, reactions of females exposed to male-conditioned water and control water, **C**, **D** - experiment no. 4, reactions of males exposed to male-conditioned water and control water. Male-conditioned water was added to the right side of the test arena while control water was introduced to the left side (*N* = 15). In the second setup (*N* = 15), the arrangement was reversed. Males and females were tested individually. MCW– male-conditioned water

In experiments 1 and 2, we tested whether there is a female-produced cue that affects *A. globator* males or females (Fig. 2A-D). In experiment 1, we assessed male responses to female-conditioned water versus control water (Fig. 2A, B). A total of 30 males were used: 15 were tested with female-conditioned water added to the right side of the arena and control water to the left, while 15 were tested in the reverse arrangement. Experiment 2 followed the same procedure with 30 females (Fig. 2C, D). In experiments 3 and 4, we tested whether there is a male-produced cue that affects *A. globator* females or males (Fig. 3A-D). In experiment 3, 30 females were tested with male-conditioned water and control water. As in experiments 1 and 2, the sides of the test arena were reversed when adding the solutions (Fig. 3A, B). Experiment 4 applied the same approach to 30 males (Fig. 3C, D).

### Statistical analyses

We examined whether males and females show a preference for male- or female-conditioned water by counting behavioral responses on the experimental and control sides of the test arena. Our null hypothesis was that mites have no preference, with responses equally likely on either side. The alternative hypothesis was that mites prefer either the experimental or control side. In all experiments, we first tested mites by adding conditioned water to the right side of the test arena, while control water was introduced to the left side (*N* = 15). In the second setup (*N* = 15), the arrangement was reversed. We used a Fisher’s test to determine if the side of the test arena influenced the males’ and females’ behavioral responses, accounting for any potential effects of uneven lighting. For both tested females and males, there were no statistically significant differences in the responses, so in subsequent analyses, the side of the test arena was not considered (*N* = 30). To investigate differences in the responses of females and males, we used a Chi-squared test or a Chi-squared test with Yates’ correction (when the expected values were less than or equal to 5). Calculations were made using PQStat software version 1.8.4 at a significance level of alpha = 0.05.

## Results

**Fig. 4:**
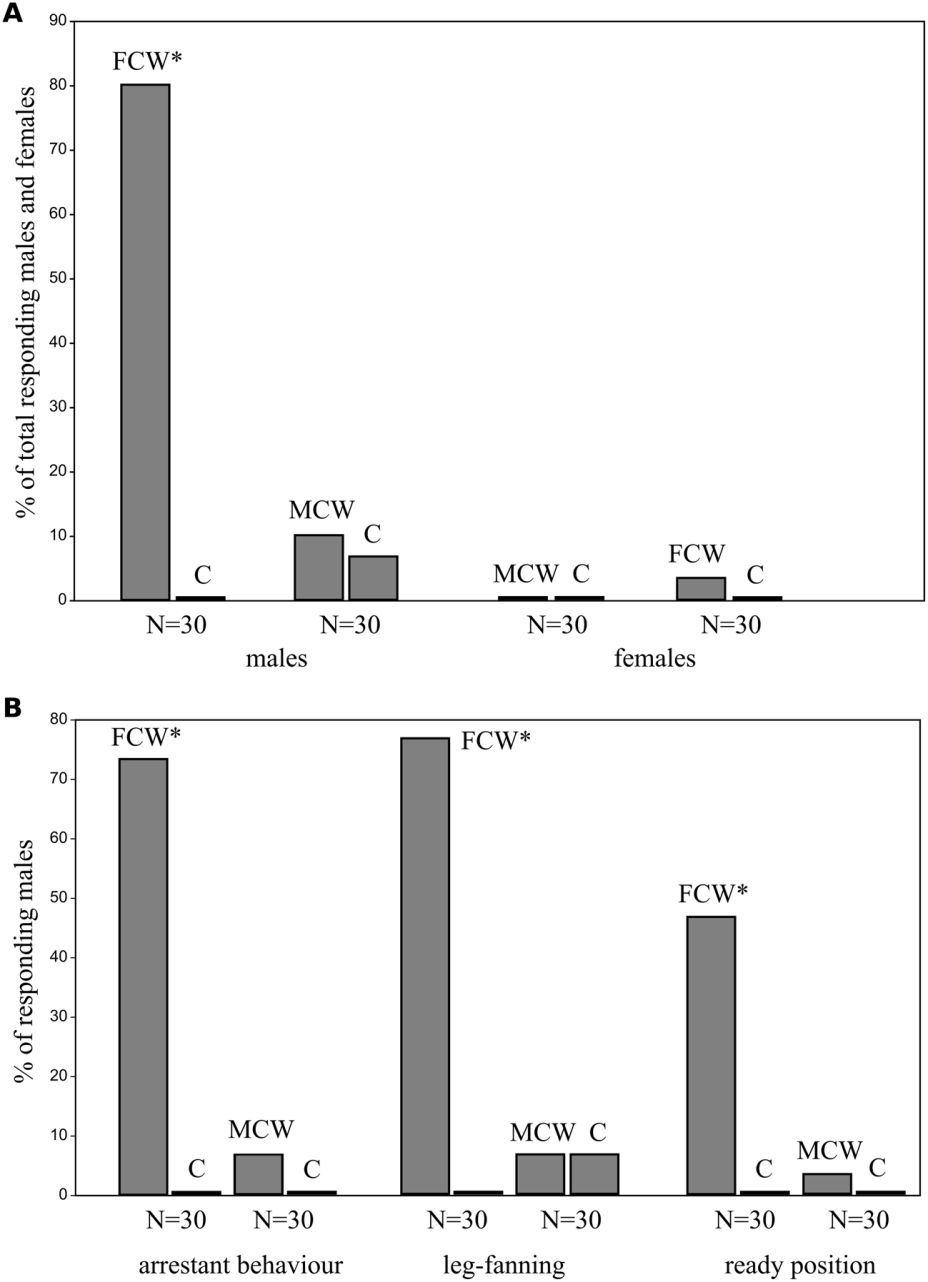
Comparison of **a**) total responding males and females *A. globator* to female-conditioned water, male-conditioned water, and control water, and **b**) behaviurs exhibited by males *A. globator* in response to female-conditioned water, male-conditioned water, and control water. As there was no significant difference in behavior due to the side of the test arena (Fisher’s test, *P* > 0.05), reactions from both sides were combined (*N* = 30). Abbreviations: FCW– female-conditioned water, MCW– male-conditioned water, C– control water. *p < 0.05

Males exhibited significant behavioral responses to female-conditioned water across all measured behaviors (arrestant behavior, leg fanning, and readiness posture), suggesting the presence of a potential female-produced sex pheromone. In total, 24 out of 30 males (80.00%) showed a response to female-conditioned water, while none did in control water. This difference is highly significant (*p* < 0.001). Specifically, 22 out of 30 males (73.33%) exhibited arrestant behavior, 23 males (76.67%) displayed leg fanning, and 14 males (46.67%) showed the readiness posture. In contrast, males did not show a significant difference in responses to male-conditioned and control water, suggesting little to no chemical communication between males (*p* = 0.999). Yet, three males (10.00%) responded to male-conditioned water, compared to two males (6.67%) that responded to control water. Arrestant behavior was exhibited by two males (6.67%) in response to male-conditioned water, with no response to control water. Leg fanning was displayed by two males (6.67%) in response to male-conditioned, compared to two males (6.67%) that responded to control water. Notably, one male (3.33%) exhibited the readiness posture in male-conditioned water, in addition to showing arrestant behavior and leg fanning, while showing no response to the control water. Unlike males, females exhibited negligible behavioral responses to either male- or female-conditioned water, indicating that potential pheromone(s) might not be as influential for females or are not detected in this setup. Female responses to both male- and female-conditioned water were not statistically significant (*p* = 0.999). Only one out of 30 females (3.33%) displayed leg fanning in response to female-conditioned water. Table 1 provides comprehensive data regarding responses from both males and females. Figure 4 visualizes the findings.

**Table 1:** Behavioral reactions of male *Arrenurus globator* to (A) female-conditioned water and control water, and (B) male-conditioned water and control water; reactions of female *Arrenurus globator* to (C) male-conditioned water and control water, and (D) female-conditioned water and control water

		Behavioral responses Number out of 30 specimens responding on the experimental side and control side of the test arena								Chi-square test
		Arrestant behavior		Leg fanning		Readiness posture		Total		
		Experimental side	Control side	Experimental side	Control side	Experimental side	Control side	Experimental side	Control side	
A	Reaction of males exposed to female-conditioned water and control water	22 (73.33%)	0 (0.00%)	23 (76.67%)	0 (0.00%)	14 (46.67%)	0 (0.00%)	24 (80.00%)	0 (0.00%)	*p* < 0.001
B	Reaction of males exposed to male-conditioned water and control water	2 (6.67%)	0 (0.00%)	2 (6.67%)	2 (6.67%)	1 (3.33%)	0 (0.00%)	3 (10.00%)	2 (6.67%)	*p* = 0.999^a^
C	Reaction of females to male-conditioned water and control water	0 (0.00%)	0 (0.00%)	0 (0.00%)	0 (0.00%)	N/A	N/A	0 (0.00%)	0 (0.00%)	*p* = 0.999^a^
D	Reaction of females to female-conditioned water and control water	0 (0.00%)	0 (0.00%)	1 (3.33%)	0 (0.00%)	N/A	N/A	1 (3.33%)	0 (0.00%)	*p* = 0.999^a^

Because there was no statistically significant difference in the behavior of males and females due to the side of the test arena (Fisher’s test, *P* > 0.05), reactions from both sides of the test arena were combined (*N* = 30); ^a^ chi-square test with Yates’ correction

## Discussion

In this study, males of *Arrenurus globator* exhibited a statistically significant response to female-conditioned water. While most males (80.00%) responded to female-conditioned water, a few (10.00%) also responded to male-conditioned water. In contrast, no response was observed from *A. globator* females to either male-conditioned or female-conditioned water. The responses of males to female-conditioned water align with the evidence reported by Smith and Hagman (2002). Although females did not exhibit behavioral responses in this study, it is likely that short-range sex pheromones or courtship behaviors play a role in intersexual communication in *Arrenurus* species (Smith and Florentino 2004).

Most males exhibited arrestant behavior and leg fanning in response to female-conditioned water, while approximately 50% displayed readiness posture. Similarly, Smith and Hagman (2002) reported that *A. manubriator* males typically exhibited arrestant behavior, with about 25% showing readiness posture. It is possible that readiness posture occurs at higher pheromonal concentrations. Additionally, other stimuli, such as physical contact with a female, may be necessary to trigger this behavior (Böttger 1962; Smith and Hagman 2002). During the initial mating phase, males and females of *Arrenurus* species sit in close proximity and touch each other with palps and forelegs before pairing (e.g., *A. manubriator*, Proctor and Smith 1994; A. *globator*, *A. cuspidator* (O.F. Müller), Więcek 2016). Since chemosensory sensilla on the palps and legs I and II have been identified in *A. acutus*, these structures are likely involved in the perception of chemical cues produced by potential mates (Baker 1996).

Unlike previous research, which primarily focused on male responses to female-conditioned water, this study examined the responses of both males and females to water conditioned by individuals of the same sex and the opposite sex. However, conclusions regarding female responses should be drawn with caution, as their behavior may vary depending on prior mating experience, and this study did not standardize the mating status of the specimens. This limitation arises because *Arrenurus* larvae, including those of *A. globator*, are temporary ectoparasites of insect hosts (Gerecke et al. 2006; Zawal 2008). Since the life cycles of mites and their hosts are synchronized, establishing laboratory colonies of mites and obtaining sufficient numbers of virgin individuals is challenging (Böttger and Martin 2003; Ramírez-Sánchez and Goldschmidt 2024).

To date, little is known about chemoreception behavior, mate recognition, and localisation of potential mates. Many questions remain unanswered and should be addressed in future research. Firstly, the exact chemical nature of the compound(s) involved in sexual attraction needs to be determined (Smith and Hagman 2002). This requires the extraction and identification of potential chemicals, followed by behavioral or electrophysiological testing of synthetic copies. In acarid mites, the female sex pheromones α-acaridial and rosefuran have been identified in *Rhizoglyphus robini* (Mizoguchi et al. 2003) and *Caloglyphus* sp. (Mori et al. 1998), respectively. Another unresolved aspect is whether potential sex pheromones are produced by dorsal glands (Böttger 1962). It is likely that they are not synthesized *de novo*, but are instead metabolites of ongoing biochemical processes, as observed in other aquatic organisms, such as shore crabs (Breithaupt and Hardege 2012).

Furthermore, the purpose of particular behaviors and dimorphic morphological structures in the possible dispersal of pheromones is unknown. An example is the role of the fanning of legs IV, which are equipped with spurs and elongated setae in the males of many *Arrenurus* species, including *A. globator* (Fig. 1A, B). Smith and Hagman (2002) suggested that the fanning of hind legs may have served to disperse the pheromones released by males of various *Arrenurus* species, as was also proposed for water mites of the genus *Neumania* (Proctor 1991, 1992). In crustaceans, stationary senders use specialized fanning structures that allow the dispersal of chemical signals (e.g., lobsters; Breithaupt and Hardege 2012). Alternatively, it is possible that leg fanning could be an adaptation for the reception of female-produced cues (Smith and Hagman 2002). Since we did not obtain any confirmation of male-produced sex pheromones in this study, we assume that the latter possibility is more likely. Finally, demographic and ecological factors impact the strength of sexual selection and eco-evolutionary dynamics in natural populations (Proctor 2003; Winkler et al. 2023). The population densities of particular water mite species can reach high values (e.g., *Piona limnetica* Biesiadka, up to 150 ind. per m³ in a lake epilimnion, see Gliwicz and Biesiadka 1975). Hence, it is unclear how chemical communication between the sexes takes place in natural populations.

## Data Availability

We declare that all data is provided within this manuscript.
